# Potential return on investment for implementation of perioperative goal-directed fluid therapy in major surgery: a nationwide database study

**DOI:** 10.1186/s13741-015-0021-0

**Published:** 2015-10-19

**Authors:** Frederic Michard, William K. Mountford, Michelle R. Krukas, Frank R. Ernst, Sandy L. Fogel

**Affiliations:** Department of Critical Care, Edwards Lifesciences, 1 Edwards Way, Irvine, CA USA; Premier Inc., Charlotte, NC USA; Current address: Quintiles, Durham, NC USA; Current address: Quintiles, Cambridge, MA USA; Current address: Indegene Total Therapeutic Management, Kennesaw, GA USA; Virginia Tech Carilion School of Medicine, Roanoke, VA USA

**Keywords:** Surgery, Complications, Costs, Goal-directed fluid therapy, Savings, Return on investment

## Abstract

**Background:**

Preventable postsurgical complications are increasingly recognized as a major clinical and economic burden. A recent meta-analysis showed a 17–29 % decrease in postoperative morbidity with goal-directed fluid therapy. Our objective was to estimate the potential economic impact of perioperative goal-directed fluid therapy.

**Methods:**

We studied 204,680 adult patients from 541 US hospitals who had a major non-cardiac surgical procedure between January 2011 and June 2013. Hospital costs (including 30-day readmission costs) in patients with and without complications were extracted from the Premier Inc. research database, and potential cost-savings associated with a 17–29 % decrease in postoperative morbidity were estimated.

**Results:**

A total of 76,807 patients developed one or more postsurgical complications (morbidity rate 37.5 %). In patients with and without complications, hospital costs were US$27,607 ± 32,788 and US$15,783 ± 12,282 (*p* < 0.0001), respectively. Morbidity rate was anticipated to decrease to 26.6–31.1 % with goal-directed fluid therapy, yielding potential gross cost-savings of US$153–263 million for the study period, US$61–105 million per year, or US$754–1286 per patient. Potential savings per patient were highly variable from one surgical procedure to the other, ranging from US$354–604 for femur and hip-fracture repair to US$3515–5996 for esophagectomies. When taking into account the volume of procedures, the total potential savings per year were the most significant (US$32–55 million) for colectomies.

**Conclusions:**

Postsurgical complications occurred in more than one third of our study population and had a dramatic impact on hospital costs. With goal-directed fluid therapy, potential cost-savings per patient were US$754–1286. The highest cost-savings per year were observed for colectomies. These projections should help hospitals estimate the return on investment when considering the implementation of goal-directed fluid therapy.

**Electronic supplementary material:**

The online version of this article (doi:10.1186/s13741-015-0021-0) contains supplementary material, which is available to authorized users.

## Background

Preventable postoperative complications are increasingly recognized as a major healthcare burden (Birkmeyer et al. 2012; Dimick et al. 2006). After major operations, especially in patients with co-morbidities, complications are not exceptions (Ghaferi et al. 2009) and have adverse effects on long-term quality of life and survival (Khuri et al. 2005; Brown et al. 2014; Artinyan et al. 2015). They are also responsible for a significant increase in hospital length of stay (LOS) (Eappen et al. 2013), readmission rates (Lawson et al. 2013; Merkow et al. 2015), and costs (Eappen et al. 2013; Dimick et al. 2004; Wick et al. 2011).

Perioperative fluid management is a key determinant of postoperative outcome. Both hypovolemia and fluid overload are associated with an increase in complications after surgery (Bellamy 2006). For a given surgical procedure, studies have shown a large intra- and inter-practitioner variability in the amount of fluid administered during the perioperative period (Lilot et al. 2015). Goal-directed fluid therapy (GDFT) consists of assessing individual fluid needs during and/or after surgery by monitoring flow parameters such as stroke volume and cardiac output (Pearse et al. 2014). A recent meta-analysis of 38 randomized controlled trials showed that the use of GDFT decreases the rate of patients developing one or more complications by 17–29 % (Pearse et al. 2014). Fuelled by this body of evidence, consensus statements (Mythen et al. 2012; Navarro et al. 2015) and guidelines (Vallet et al. 2013; Gustafsson et al. 2013) have been published, and GDFT has been integrated into the Enhanced Recovery in NSQIP (ERIN) collaborative. But today, the adoption of GDFT is still very limited in the USA (Cannesson et al. 2011; Miller et al. 2011). One of the barriers to the hospital adoption of GDFT may be the short-term financial investment necessary to acquire cardiac output-monitoring technologies. To ensure a fair economic evaluation, this investment must be balanced with the economic benefits related to the decrease in complications that is expected from the implementation of GDFT.

The goals of our study were twofold: describe the economic consequences of postoperative complications in a nationwide population of patients undergoing major non-cardiac surgery and estimate the economic impact of the reduction in postoperative morbidity expected from GDFT. This estimation should help hospitals to estimate the return on investment when considering the implementation of GDFT.

## Methods

### Data source

De-identified data from all adult inpatients, who had major non-cardiac surgery between January 2011 and June 2013, were selected from the Premier research database. The Premier research database contains patient data from over 600 US hospitals spanning all geographic regions, containing teaching and non-teaching as well as urban and rural hospitals of all sizes. Examination of the database allows the determination of patients’ characteristics, postsurgical complications, and costs of care. The database complies with the Health Insurance Portability and Accountability Act of 1996 (HIPAA) and other related regulations. Any institutional review board approval was not sought because of the pre-existing, retrospective, and de-identified nature of the data.

### Patient selection

Ten major surgical procedures were selected based on previous studies showing positive outcomes associated with the use of GDFT (Benes et al. 2010; Bisgaard et al. 2013; Boyd et al. 1993; Gan et al. 2002; Kuper et al. 2011; Lobo et al. 2000; Noblett et al. 2006; Pearse et al. 2005; Pillai et al. 2011; Ramsingh et al. 2013; Sinclair et al. 1997; Ueno et al. 1998; Venn et al. 2002; Wakeling et al. 2005; Wilson et al. 1999). Corresponding International Classification of Diseases, Ninth Revision, Clinical Modification (ICD-9) codes were used to search specific procedures in the Premier research database (see Additional file 1: Table S1). Because GDFT has thus far been shown to be effective only in adults, those under 18 years of age were excluded. Patients in whom cardiac output was monitored on the day of surgery were also excluded, since they may have received GDFT.

### Patient characteristic data collection

Patients’ characteristics, including gender, age, and co-morbidities (based on ICD-9 diagnosis codes) were tabulated. The Charlson Co-morbidity Index (CCI) was calculated as previously described (Charlson et al. 1994). Twenty six infectious, gastro-intestinal, respiratory, renal, cardiovascular, neurologic, and hematologic in-hospital postoperative complications were identified using ICD-9 diagnosis codes, ensuring that the diagnosis was not determined to be present at admission (see Additional file 2: Table S2). Morbidity rate was defined as the proportion of patients developing one or more complications during the index hospital stay. Patients were sorted into two groups: those with one or more complications and those without any complications. For each group, hospital length of stay and readmission rates at 30 days were studied.

### Cost data collection and cost-savings projection

Costs related to the in-hospital treatment and readmission up to 30 days after discharge were obtained from the Premier database and compared in patients with and without complications. Costs are those associated with the actual procedures, as determined by the hospital using its accounting systems, and include both fixed and variable components.

The recently published JAMA meta-analysis by Pearse et al. (Pearse et al. 2014) was used to estimate the potential reduction in postoperative morbidity with GDFT. This meta-analysis reported an average 23 % reduction in odds of a postoperative complication (odds ratio 0.77, 95 % CI 0.71 to 0.83, *p* < 0.05) associated with the use of GDFT. Potential cost-savings were determined by using the projected number of patients developing one or more complications and the estimated related costs. This analysis was performed for the entire cohort, as well as for each surgical procedure. The analysis assumes a complete, new implementation of GDFT.

### Statistical analysis

Hospital LOS, readmission rates, and costs were compared between patients with and without complications. Readmission rates (%) were compared using chi-squared tests and hospital LOS (median ± interquartiles), and costs (mean ± SD) were compared using Wilcoxon rank-sum and *t* tests, respectively. All statistical comparisons were considered statistically significant assuming a two-tailed alpha level of 0.05.

Total costs were further analyzed using multivariable generalized linear models. The models utilized a gamma distribution and log link to account for the skewness in the outcome data. The model estimated the least-squares mean total costs while controlling for potential confounding variables including patient age, gender, co-morbidities (as measured by the CCI), and elective/non-elective admission. An additional model was performed as a sensitivity analysis and added the type of surgery to the other potential confounders.

## Results

**Fig. 1:**
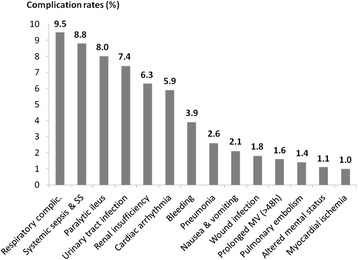
Complication rates

A total of 204,680 patients from 541 medical centers met the search criteria. Among these centers, 86.6 % were urban, 58.3 % were non-teaching hospitals, and 65.7 % had 300 or more beds. Patients’ characteristics are summarized in Table 1. Numbers of patients per surgery group are reported in Table 2. A total of 76,807 patients developed one or more postsurgical complications (average morbidity rate 37.5 %). Complication rates ≥1 % are shown in Fig. 1. Morbidity rates for each surgery group are presented in Table 3.

**Table 1:** Characteristics of the study population

	All	With complications	Without complications
	*n* = 204,680	*n* = 76,807	*n* = 127,873
Age (years)	64.8 ± 17.2	68.8 ± 16.0	62.4 ± 17.4
Gender (% female)	58.8	58.4	59.0
Elective surgery (%)	54.8	45.2	60.5
ICU admission (%)	22.4	36.2	14.1
Mortality (%)	1.9	4.6	0.2
Myocardial infarction (%)	6.2	8.8	4.6
Congestive heart failure (%)	7.5	12.3	4.6
Peripheral vascular disease (%)	7.3	9.0	6.2
Cerebrovascular disease (%)	2.0	3.2	1.4
Hemiplegia or paraplegia (%)	0.4	0.7	0.2
Dementia (%)	0.8	1.1	0.5
Chronic pulmonary disease (%)	19.3	23.8	16.6
Rheumatologic disease (%)	2.6	3.0	2.3
Peptic ulcer disease (%)	1.3	1.9	0.9
Mild liver disease (%)	1.0	1.3	0.8
Moderate or severe liver disease (%)	0.4	0.6	0.2
Diabetes (%)	19.9	21.1	19.2
Diabetes with complications (%)	2.2	2.8	1.9
Renal disease (%)	9.3	14.1	6.3
Any malignancy (%)	22.1	24.1	20.8
Metastatic solid tumor (%)	6.6	7.7	6.0
AIDS (%)	0.1	0.1	0.1
Charlson Co-morbidity Index	1.8 ± 2.3	2.2 ± 2.5	1.5 ± 2.2

All comparisons “with complications vs. without complications” were statistically significant with *p* < 0.0001, with the exception of gender (*p* = 0.0386) and AIDS (*p* = 0.2912)

**Table 2:** Hospital length of stay (HLOS), 30-day readmission rate, and costs in patients with one or more complications (with) and in patients without any complications (without)

Surgery	HLOS, days, median [IQR]		Readmission rate, %		Cost, dollar^a^, mean ± SD	
*n*	With	Without	With	Without	With	Without
All	7 [4–10]	4 [3–5]	17.2	11.9	27,607 ± 32,788	15,783 ± 12,282
204,680						
AAA open repair	8 [6–14]	6 [4–7]	16.5	8.7	48,002 ± 48,841	24,619 ± 14,543
2328						
Vascular bypass	6 [4–9]	3 [2–5]	21.3	14.1	31,979 ± 30,386	16,849 ± 12,543
16,336						
Esophagectomy	13 [9–20]	9 [8–11]	18.5	15.4	67,924 ± 65,377	37,382 ± 17,973
690						
Gastrectomy	4 [2–10]	2 [1, 2]	12.7	5.2	27,794 ± 33,530	12,641 ± 9,452
25,118						
Colectomy	8 [5–11]	4 [3–6]	15.2	9.0	27,851 ± 29,286	14,755 ± 10,524
75,121						
Resection of rectum	7 [5–11]	5 [3–6]	15.2	10.4	26,916 ± 24,466	15,979 ± 18,855
10,753						
Hepatectomy	7 [5–11]	5 [3–6]	17.9	9.4	37,315 ± 38,100	20,272 ± 13,566
2362						
Pancreatectomy	11 [8–18]	7 [5–9]	26.1	18.6	50,559 ± 46,784	27,488 ± 19,653
3569						
Cystectomy	10 [7–14]	7 [6–8]	29.2	21.9	41,128 ± 38,293	25,978 ± 15,061
2552						
F&H fracture repair	5 [4–7]	4 [3–5]	18.9	17.4	22,218 ± 32,644	16,805 ± 12,167
65,851						

All comparisons “with vs. without” were statistically significant with *p* < 0.0001

*AAA* abdominal aortic aneurysm, *F&H* femur and hip

^a^For patients with valid cost data, unadjusted

**Table 3:** Morbidity rate, cost difference between patients with and without complications, and expected savings per patient receiving goal-directed fluid therapy (GDFT)

Surgery	Morbidity rate, %	Cost difference between patients with and without complications, dollar	Potential savings per patient with GDFT, dollar^a^
All	37.5	11,824	754–1286
AAA open repair	64.9	23,383	2580–4401
Vascular bypass	26.3	15,130	676–1154
Esophagectomy	67.7	30,542	3515–5996
Gastrectomy	20.2	15,153	520–888
Colectomy	43.3	13,096	964–1644
Resection of rectum	33.6	10,937	625–1066
Hepatectomy	34.3	17,043	994–1695
Pancreatectomy	47.5	23,071	1863–3178
Cystectomy	58.9	15,150	1517–2588
F&H fracture repair	38.5	5413	354–604

*AAA* abdominal aortic aneurysm, *F&H* femur and hip

^a^For patients with valid cost data, unadjusted

### Impact of postsurgical complications on hospital length of stay and readmission rate

In patients with one or more complications and in patients without any complications, 30-day readmission rates were 17.2 and 11.9 % (*p* < 0.001), respectively. Median hospital length of stay was 7 [4, 10] (25th–75th percentiles) and 4 [3, 5] days (*p* < 0.001), respectively. The impact of postoperative complications on hospital length of stay and 30-day readmission rates for each surgery group is presented in Table 2.

### Economic impact of postsurgical complications

Average unadjusted costs (index hospitalization + 30-day readmission costs when applicable) were US$27,607 ± 32,788 per patient with one or more complications (*n* = 73,108) and US$15,783 ± 12,282 (*p* < 0.001) per patient with no complications (*n* = 127,398). In other words, patients with one or more complications were on average US$11,824 more costly than patients with no complications. Thus, from January 2011 to June 2013, the 541 hospitals whose data were used in these analyses spent an estimated total of more than US$908 million (US$11,824 × 76,807 patients) to treat postsurgical complications in the study population (US$363 million per year). The economic impact of postoperative complications for each surgery group is presented in Table 2.

Results from the multivariable model were directionally consistent with the descriptive analysis. The average estimated costs after controlling for confounders were significantly different, US$25,390 vs. US$14,841 (*p* < 0.001, cost difference US$10,549) per patient with one or more complications and per patient with no complications, respectively. Furthermore, when the model also controlled for the type of surgery, the estimated costs remained significantly different (US$32,182 vs. $19,803; *p* < 0.001, cost difference US$12,379).

### Projected cost-savings with implementation of GDFT

**Fig. 2:**
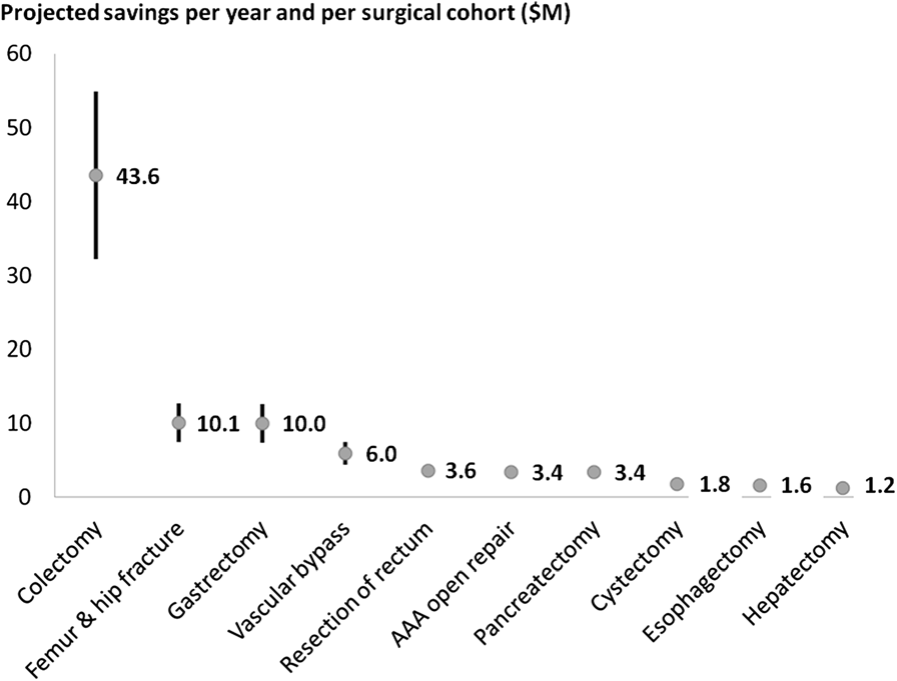
Projected cost-savings per year and per surgical cohort. The numerical value is the mean in million dollars. Each *vertical bar* represents the range between minimum and maximum savings related to a 17 or 29 % decrease in postoperative morbidity, respectively

The projected number of patients developing one or more complications, assuming an odds ratio ranging between 0.71 and 0.83, was 54,533–63,750 (morbidity rate 26.6–31.1 %). Thus, after implementation of GDFT, projected gross savings were US$153–263 million for the study period, US$61–105 million per year, or US$754–1286 per patient (Table 3). Projected cost-savings per year for each surgery group are presented in Fig. 2.

## Discussion

In our large patient population who underwent major non-cardiac surgery, postoperative complications were observed in more than one third of the cases and increased costs on average by US$11,824 per patient (+75 %). These findings are consistent with those reported by previous and smaller studies. In 1008 patients who underwent general and vascular surgery, Dimick et al. (Dimick et al. 2006) reported a US$10,178 cost difference between patients with and without complications. In a similar surgical population, Boltz et al. (Boltz et al. 2012) showed in 2250 patients that the excess costs were US$6358, US$12,802, and US$42,790 for patients developing 1, 2, 3 or more complications, respectively. In the present study, the occurrence of postoperative complications was also associated with prolonged length of stay (+3 days) and increased hospital readmission rates at 30 days (+5.3 % absolute increase, +44 % relative increase). These findings are consistent with previous reports (Eappen et al. 2013; Lawson et al. 2013) emphasizing the dramatic impact of complications on length of stay and readmission rates. This highlights a relevant savings capacity for major surgical procedures.

According to a recent meta-analysis of 38 randomized controlled trials, GDFT has the potential to decrease postoperative morbidity by 17–29 % (Pearse et al. 2014). In our study population, such a decrease in postoperative morbidity would translate into cost-savings ranging between US$754 and US$1286 per patient. Interestingly, potential savings were highly variable from one surgical procedure to the other (Table 3). Two factors affect savings per patient: the actual morbidity rate (the higher the morbidity rate, the higher the savings when all patients receive GDFT) and the cost of complications (the higher the cost, the higher the savings). The actual morbidity rates ranged from 20.2 % for gastrectomies to 67.7 % for esophagectomies (Table 3), and cost of complications ranged from US$5413 for femur and hip-fracture repair to US$30,542 for esophagectomies (Table 3). This large range in morbidity rates and costs of complications explain why the range of potential savings with GDFT was also wide, from US$354–604/patient for femur and hip-fracture repair to US$3515–5996/patient for esophagectomies (Table 3). A third factor affects savings at the hospital level: the volume of surgeries. When taking into account the volume of procedures, the total potential savings per year were the most significant (US$32–55 million), for colectomies, by far (Fig. 2). This finding supports the notion that GDFT should be implemented as a priority in this surgical population.

Three recent studies tried to estimate potential savings related to the use of GDFT. The first study (Bartha et al. 2012) from Sweden is a decision analytic model where assumptions were made regarding morbidity rates before and after GDFT implementation, as well as on hospital costs. This study focused on elderly hip-fracture patients, and the model estimated a 1882 € (around US$2000) cost reduction per patient with GDFT. The second study from the UK was based on a small population of 122 patients who underwent major surgery (Ebm et al. 2014). Morbidity rates before and after GDFT implementation were real, but assumptions were made regarding hospital costs. This study suggested a cost reduction of £2631 (around US$4000) per patient with GDFT. Differences between US and European healthcare systems and costs, as well as the fact that costs were not real but estimated in the Swedish and the UK study, may explain why they both reported potential savings higher than our projections. In the third study, Manecke et al. (Manecke et al. 2014) used real morbidity rates and real costs extracted from the UHC database, which is a large administrative database containing clinical and economic data from over 120 US academic hospitals. As in our study, the only assumption made was related to the reduction in postoperative morbidity with GDFT. Manecke et al. (Manecke et al. 2014) reported potential cost-savings ranging from US$569 to US$970 per patient, i.e., slightly lower than ours. The UHC database contains only 11 possible postoperative complications and is known to underestimate postoperative morbidity rates (Steinberg et al. 2008). For instance, they did not take into account postoperative paralytic ileus, which is not a major complication but a frequent one (Fig. 1), known to have a significant impact on hospital length of stay and costs (Iyer et al. 2009). Finally, the economic evaluation of Manecke et al. (Manecke et al. 2014) did not include 30-day readmission costs, and was limited to academic hospitals. Our analysis was based on a larger number of patients and considered 26 different postoperative complications (including paralytic ileus). We took into account 30-day readmission costs, and 58.2 % of our study population came from non academic centers. For these reasons, we believe that our study provides a more accurate estimation of potential savings associated with the implementation of GDFT at a national level.

To assess a return of investment, our projected savings must be balanced with costs related to GDFT implementation. Assuming average cardiac output-monitoring-related costs of US$300 per patient (US$250 for disposable sensor + US$48 for the amortization of a US$15,000 monitor used two times a week over 3 years), our findings suggest that for each dollar spent to implement GDFT, hospitals should save in return between US$2.5 and US$4. GDFT implementation costs may vary from one hospital to the other, but each hospital could easily forecast the return on investment using its own costs and our model.

Another major burden of complications is the opportunity cost of lost beds for increased length of stay and re-admissions in patients with complications. In busy hospitals, these are beds not taken by new patients with new DRGs and the accompanying payments. With an increased length of stay of 3 days for patients with complications (Table 2), the 76,807 patients with complications represent 230,421 (3 × 76,807) days lost. With the assumption that GDFT would decrease the number of patients with one or more complications to 54,533–63,750 (a 17 to 29 % decrease), it would now represent only 163,599–191,250 days lost. In other words, GDFT has the potential to save between 39,171 and 66,822 days. With the average hospital length of stay across all studied surgical cohorts being 5 days, the implementation of GDFT could result in 7834–13,364 (39,171 and 66,822 divided by 5) new patients admitted in our 541 hospitals over the 2.5-year study period, or 3134–5346 new patients per year. Since payments to hospitals vary so widely, each hospital can use this approach to calculate the increased payment and profit to the bottom line. This is the lost opportunity cost of having patients with complications take up hospital beds needed for other patients and represents the potential additional profit to the hospital. This may easily outweigh the savings from decreased complications.

Our study has certain limitations that should be considered. The analysis was limited to specific major surgeries in which outcome has already conclusively been shown to be improved by the use of GDFT. There are other surgeries, such as major spine and gynecologic surgeries, in which this approach would likely be associated with fewer complications (Mythen et al. 2012; Gan et al. 2002). Our study assumes complete implementation of GDFT, which may be an unrealistic goal. Also, we considered the same postsurgical morbidity reduction with GDFT for all surgical procedures, which may not always be the case. However, previous meta-analysis (Pearse et al. 2014; Hamilton et al. 2011) did not find any interaction between the type of surgery and the effect of GDFT. The article by Pearse et al. (Pearse et al. 2014) was chosen to estimate the effect of GDFT on postoperative morbidity because it is the most recent meta-analysis on the topic. It is important to note that it included studies published many years ago. Because both anesthesia and surgical practices changed over time, the assumption that GDFT would reduce postoperative morbidity by 17–29 % may be questioned in 2015. A meta-analysis by Hamilton et al. (Hamilton et al. 2011) studied the clinical effects of GDFT over time. If GDFT had no effect on mortality in studies published after 2000, the reduction in postoperative morbidity was still highly significant, ranging between 50 and 71 % (average odds ratio 0.38, CI 0.29 to 0.50). Interestingly, if we had used these odds ratios for our calculations, the projected savings with GDFT would have been much higher than those we have reported. Having said that, we must acknowledge that enhanced recovery programs have gained acceptance only recently, as well as changes in the type of fluid administered during the perioperative period (starches and unbalanced crystalloid solutions are used less often). Therefore, the clinical effects of GDFT in this new perioperative medicine era remain to be evaluated by large studies. Finally, we did not have access to reimbursement data, so we were unable to study the effects of morbidity reduction on hospital profit or profit margin, another very important economic driver for the hospital adoption of any new therapeutic strategy (Dimick et al. 2006; Flynn et al. 2014).

## Conclusions

In patients who underwent major non-cardiac surgery, our study demonstrates that postsurgical complications are frequent and have a significant impact on hospital length of stay, readmission rates, and costs. It also suggests significant savings with GDFT; for each dollar spent to implement GDFT, our projections suggest that hospitals should save in return between US$2.5 and US$4. Projected cost-savings were the highest for the colectomy cohort, suggesting that priority should be given to the implementation of GDFT in this patient population.

## Additional files

Additional file 1:
**The 10 major surgical procedures, with the corresponding ICD-9 procedure codes.** DOC 72.5 kb Additional file 2:
**The 26 postsurgical complications, with the corresponding ICD-9 diagnostic codes.** DOC 58.0 kb

## Abbreviations

GDFT: Goal-directed fluid therapy
CCI: Charlson co-morbidity Index
