# Supplementary material for: Potential return on investment for implementation of perioperative goal-directed fluid therapy in major surgery: a nationwide database study
Source: Perioper Med (Lond). 2015 Oct 19;4:11. doi: 10.1186/s13741-015-0021-0 (PMC4615879; doi:10.1186/s13741-015-0021-0)
Supplement: Additional file 1: — The 10 major surgical procedures, with the corresponding ICD-9 procedure codes. DOC 72.5 kb [file 13741_2015_21_MOESM1_ESM.doc]

**Additional file 1: Table S1. The 10 major surgical procedures, with the corresponding ICD-9 procedure codes.**

| **Surgical procedure** | **ICD9 procedure codes** |
| --- | --- |
| **VASCULAR** |  |
| Abdominal aortic aneurysm open repair | 38.44 |
| Aorto-iliac & peripheral bypass | 39.25, 39.29 |
| **GASTRO-INTESTINAL** |  |
| Esophagectomy | 42.40, 42.41, 42.42 |
| Gastrectomy | 43.5, 43.6, 43.7, 43.81, 43.89, 43.91, 43.99 |
| Colectomy | 45.71-45.76, 45.79, 45.81-45.83 |
| Resection of rectum | 48.40, 48.43, 48.49-48-52, 48.59, 48.61-48.65, 48.69 |
| Hepatectomy | 50.22, 50.3 |
| Pancreatectomy | 52.51-52.53, 52.59, 52.6, 52.7 |
| **UROLOGIC** |  |
| Total cystectomy | 57.71, 57.79 |
| **ORTHOPEDIC** |  |
| Femur & hip fracture repair | 79.15, 79.25,79.35,79.85,79.95 |
