# Supplementary material for: Potential return on investment for implementation of perioperative goal-directed fluid therapy in major surgery: a nationwide database study
Source: Perioper Med (Lond). 2015 Oct 19;4:11. doi: 10.1186/s13741-015-0021-0 (PMC4615879; doi:10.1186/s13741-015-0021-0)
Supplement: Additional file 2: — The 26 postsurgical complications, with the corresponding ICD-9 diagnostic codes. DOC 58.0 kb [file 13741_2015_21_MOESM2_ESM.doc]

**Additional file 2: Table S2. The 26 post-surgical complications, with the corresponding ICD-9 diagnostic codes.**

| **Post-surgical complication** | **ICD9 diagnosis codes** |
| --- | --- |
| **INFECTION** |  |
| Pneumonia | 482.xx,484.x,485,486,997.31,997.32 |
| Urinary tract infection | 590.xx,595.xx,599.0x,996.64 |
| Wound infection | 996.6x (except 996.64),998.5x |
| System sepsis/septic shock | 038.xx,566,567.xx,569.5,682.x,686.xx,785.52,995.91,  995.92 |
| **GASTRO INTESTINAL** |  |
| Nausea & vomiting | 536.2,564.3,787.0x |
| Ileus (paralytic or functional) | 560.1,564.4 |
| Acute bowel obstruction | 560.xx (except 560.1) |
| Gastro-intestinal bleeding | 578.x |
| Abdominal compartment syndrome | 729.73 |
| Hepatic dysfunction | 570,573.3 |
| Pancreatitis | 577.0 |
| **RESPIRATORY** |  |
| Prolonged mechanical ventilation (>48h) | 96.70^a^,96.71^a^,96.72 |
| Respiratory failure or ARDS | 514,518.4,518.5x,518.81,518.84,786.0x,799.0x |
| Pleural effusion | 511;1,511.8x,511.9 |
| **RENAL** |  |
| Renal insufficiency or failure | 593.9,584.x,586,788.5 |
| **CARDIOVASCULAR** |  |
| Deep venous thrombosis | 451.xx,452,453.xx |
| Pulmonary embolism | 415.1x |
| Myocardial ischemia or infarction | 410.xx,411.xx,413.x |
| Arrhythmia | 427.xx,785.1,997.1 |
| Shock | 785.51,998.0x |
| Infarction of GI tract | 557.0 |
| **NEUROLOGY** |  |
| Stroke | 431,432.x,433.xx,434.xx,435.x,436,437.x |
| Coma | 780.0x |
| Altered mental status | 293.0,293.1,438.0,780.97,799.5x |
| **HEMATOLOGY** |  |
| Bleeding requiring transfusion | 99.0x^b^ |
| **OTHER** |  |
| Wound dehiscence | 998.3x |

^a^ code must be present for 2 or more consecutive days,

^b^ 1 or more units of red blood cells or whole blood on any day after day of surgery.
